# Supplementary material for: Control of metal oxides’ electronic conductivity through visual intercalation chemical reactions
Source: Nat Commun. 2023 Oct 2;14:6130. doi: 10.1038/s41467-023-41935-x (PMC10545781; doi:10.1038/s41467-023-41935-x)
Supplement: Supplementary file 1 — Supplementary Information [file 41467_2023_41935_MOESM1_ESM.pdf]

Supplementary Information

Control of Metal Oxides' Electronic Conductivity Through Visual Intercalation Chemical Reactions

Yuanyuan Zhang et al.

Supplementary Figures

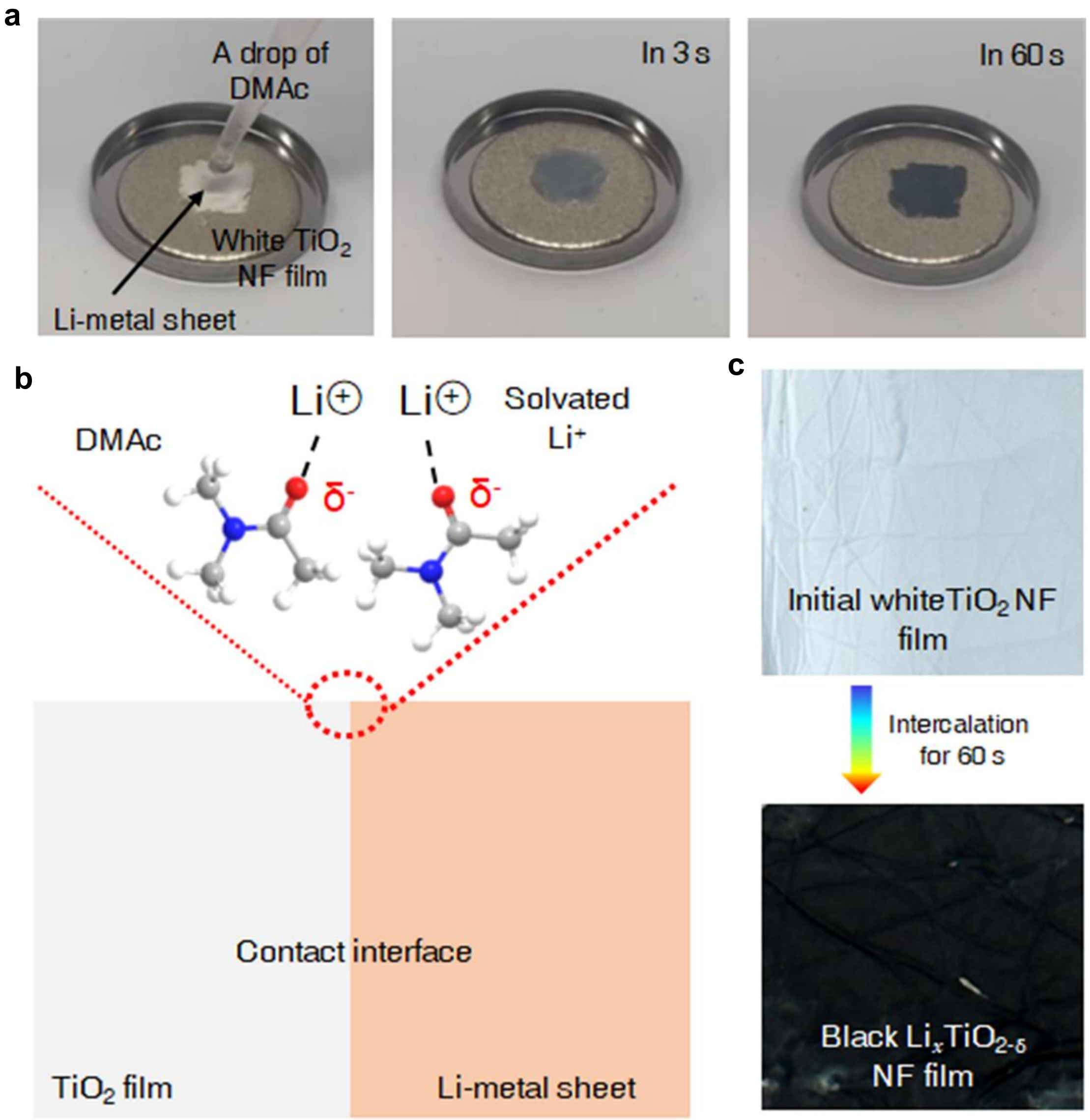

Supplementary Fig. 1. Illustrations of model 3 applied in this study. (**a**) Visualization of the quicky color changes of the white TiO_2 NF film after contacting with a Li-metal sheet. (**b**) Demonstration of the contact-reduction model for a Li-metal and a TiO_2 NF film. (**c**) Digital photos of the TiO_2 NF films before and after the intercalation in model 3, the white film turned black in 1 min.

According to Gibbs function, Li-metal can spontaneously reduce TiO_2 at room temperature, but the spontaneous reaction only happens when they contact. Here, DMAc was selected as a diffusion solvent due to its high wettability with the oxide films and the electron-pulling ability. The self-driven chemical reaction (interfacial contact corrosion reaction) induces oxygen-defects and initiates an interfacial insulation-to-conduction phase transition of the interfacial TiO_2 NFs, which triggers a rapid reduction from the interface to the whole TiO_2 NF film and the film is quickly changed from white to black in 1 min. It is well known that electron transfer will occur on the contact surface due to their different electric Fermi levels (E_F). According to the work function ($W = E_0 - E_F$), Li has lower W than TiO_2 due to its higher E_F. Therefore, Li will donate electrons to TiO_2 and TiO_2 was first reduced at the interface. Such self-driven chemical reactions will continue if there is a Fermi level difference, and the contact-corrosion creates lots of Li^+-ions or Li-NPs. The conductive interfacial layer works as a bridge and delivers electrons to the adjacent nonconductive TiO_2 layers continuously. At the same time, the unstable and active solvated Li^+-ions or NPs, which transfer quickly from the interface to the whole film due to the micro-electrical fields and the strong siphon effect of the nanofibrous structures, are ready to react with the deprived oxygen-atoms. This smooth flow of electrons and the diffusion of lithium led to the layer-by-layer creation of OVs in the whole NF film.

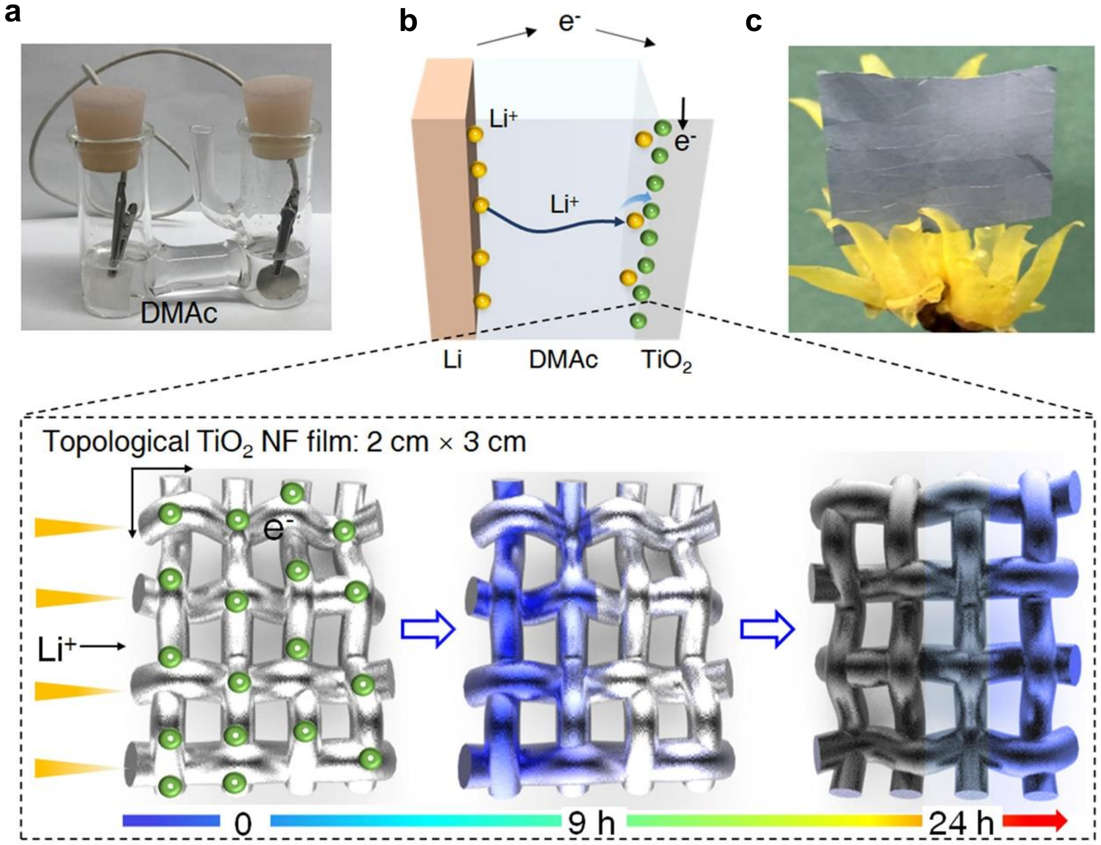

Supplementary Fig. 2. Demonstration of the color evolution in the white TiO_2 NF films in model 1. (**a**) The practical model used in this study. A piece of white TiO_2 NF film (2 cm × 3 cm) and a piece of circular Li-sheet (d=2.1 cm) were first vertically and outwardly immersed into DMAc in an H-type beaker, and then were connected by a wire on the top of these two films. Here, the two films were not in contact, and the solvents could flow through the middle bridge channel at both ends. (**b**) Demonstration of the intercalation pathways along the Li+-ion diffusion, and the corresponding color changes from white to blue and then to black of the TiO_2 NF film from the left to the right. (**c**) Optical photograph of a blue $Li_xTiO_{2-\delta}$ NF film treated by model 2 after 12h.

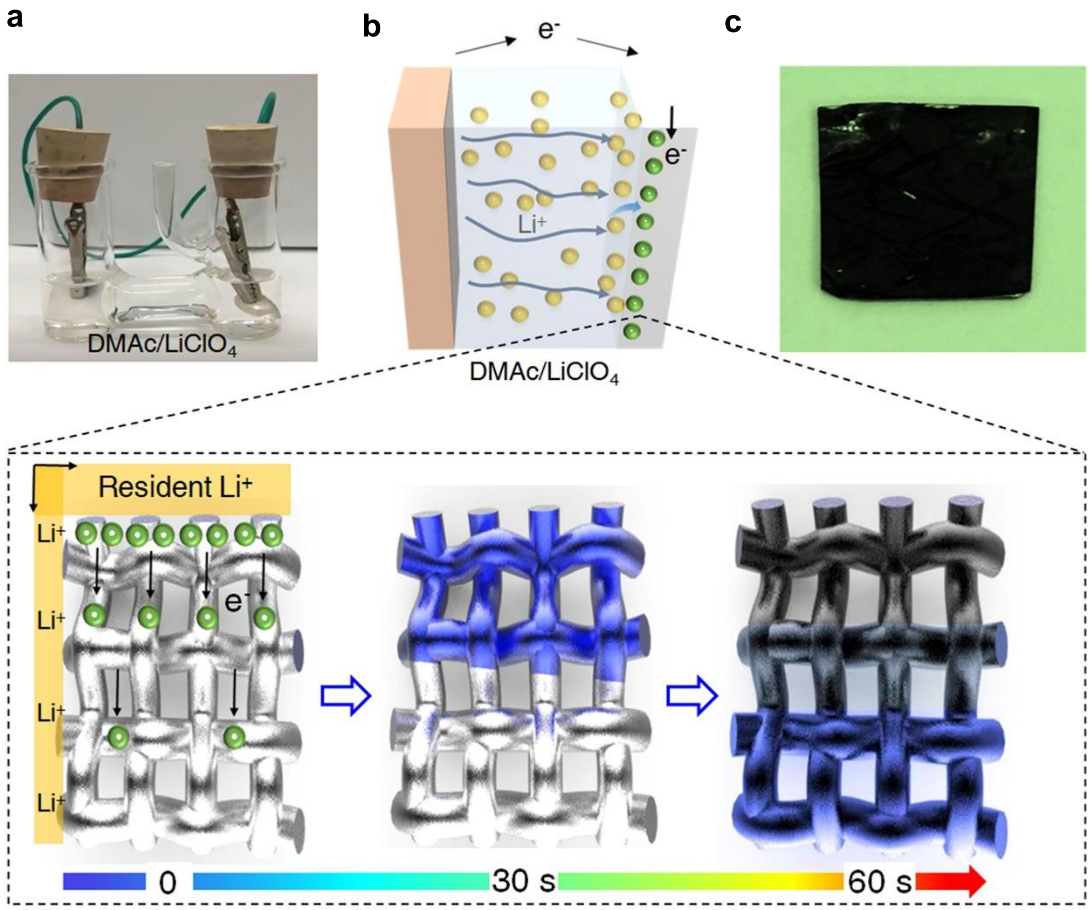

Supplementary Fig. 3. Demonstration of the color evolution in the white TiO_2 NF films in model 2. (**a**) The practical model used in this study. The device used is the same with model 2, but the solvent was a mixture of DMAc/$LiClO_4$ in an H-type beaker. (**b**) Demonstration of the intercalation pathways along the electron conduction, and the corresponding color changes from white to blue and then to black of the TiO_2 NF film from top to down. (**c**) Optical photograph of a black $Li_xTiO_{2-\delta}$ NF film treated by model 3 after 3 minutes.

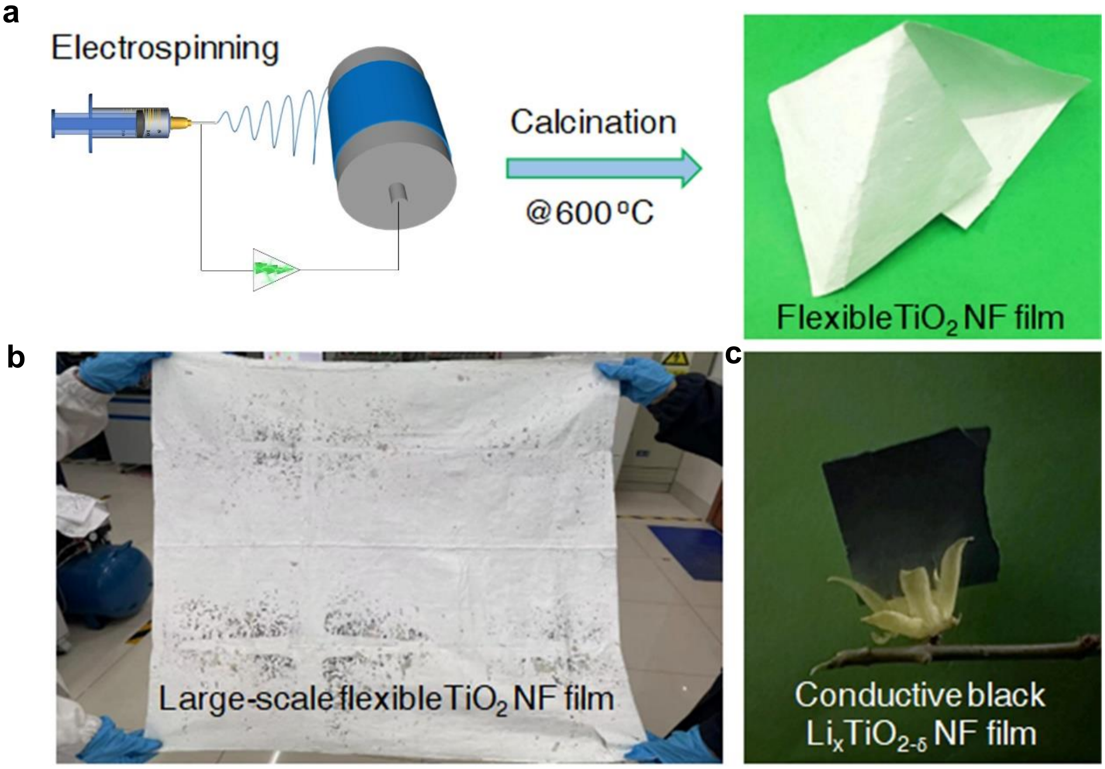

Supplementary Fig. 4. The preparation of flexible TiO_2 NF film. (**a**) Schematic diagram of fabricating flexible TiO_2 NF film by electrospinning in our lab. The porosity and NF diameter could be finely tuned by adjusting the applied voltage, the viscosity and conductivity of the sol, as well as the transfer distance between the needle and the receiving plate. Generally, the electrospun TiO_2 NF film had a porosity of >80%. (**b**) A large piece of flexible TiO_2 NF film fabricated with a pilot production machine. (**c**) A flexible and conductive black $Li_xTiO_{2-\delta}$ NF film fabricated by the topochemical synthetic strategy.

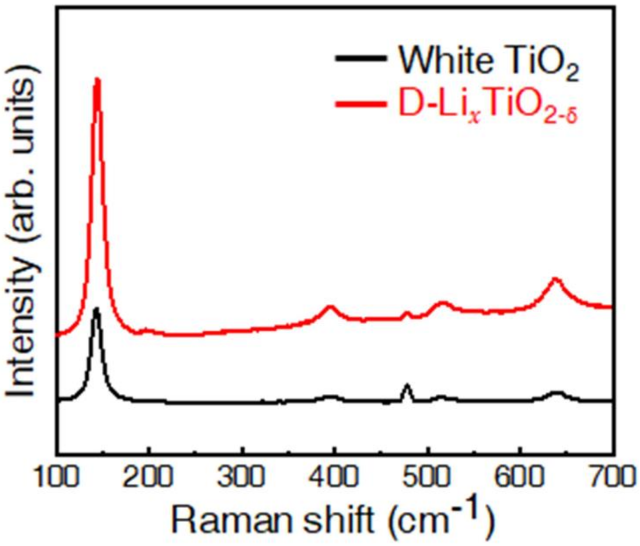

Supplementary Fig. 5. Raman characterization of the initial white TiO_2 NFs and the intercalated D-$Li_xTiO_{2-\delta}$ NFs. The Raman spectra of D-$Li_xTiO_{2-\delta}$ NFs showed a similar configuration with the initial TiO_2 NFs, but a clear blue-shift of peak and a broadened E_g peak were observed, further confirming the robust intercalated $Li_xTiO_{2-\delta}$ structure and the structural evolution by nonstoichiometric measurements.

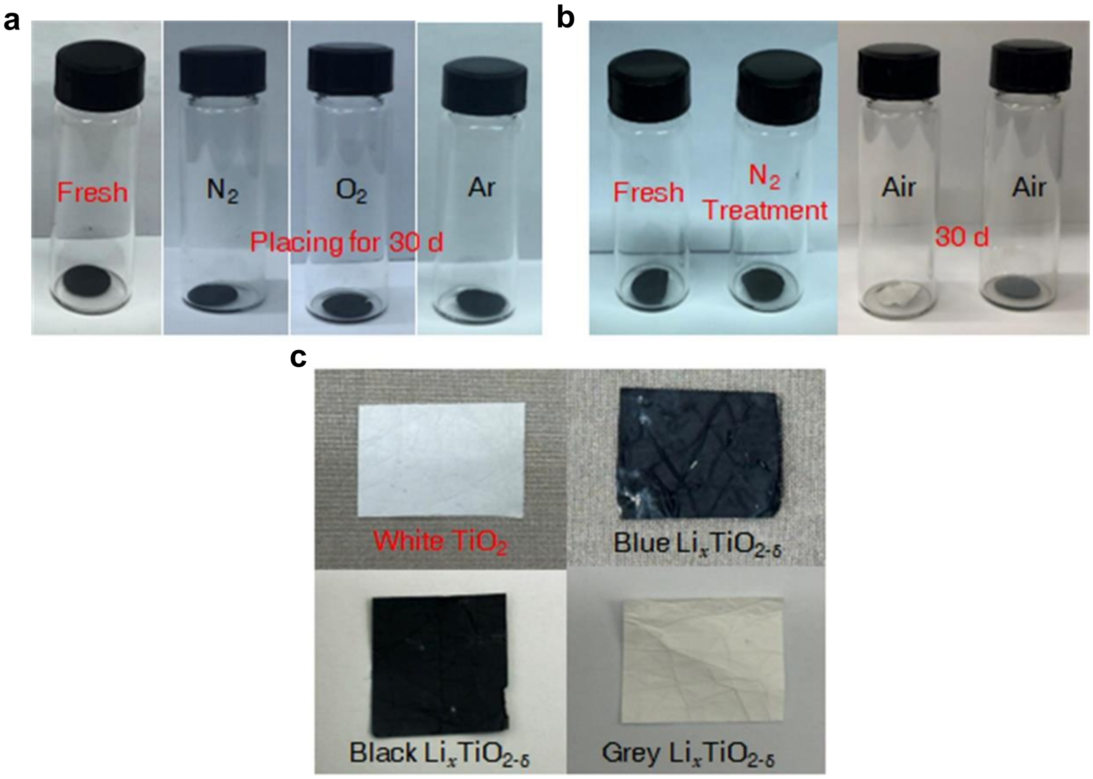

Supplementary Fig. 6. Stability characterizations of the intercalated $Li_xTiO_{2-\delta}$ NF films. (**a-b**) Digital photos of the prepared fresh black $Li_xTiO_{2-\delta}$ films and their color changes after 30 d in different environmental conditions. (**c**) Visualization of the color evolution of the initial white TiO_2 NF film treated with model 3.

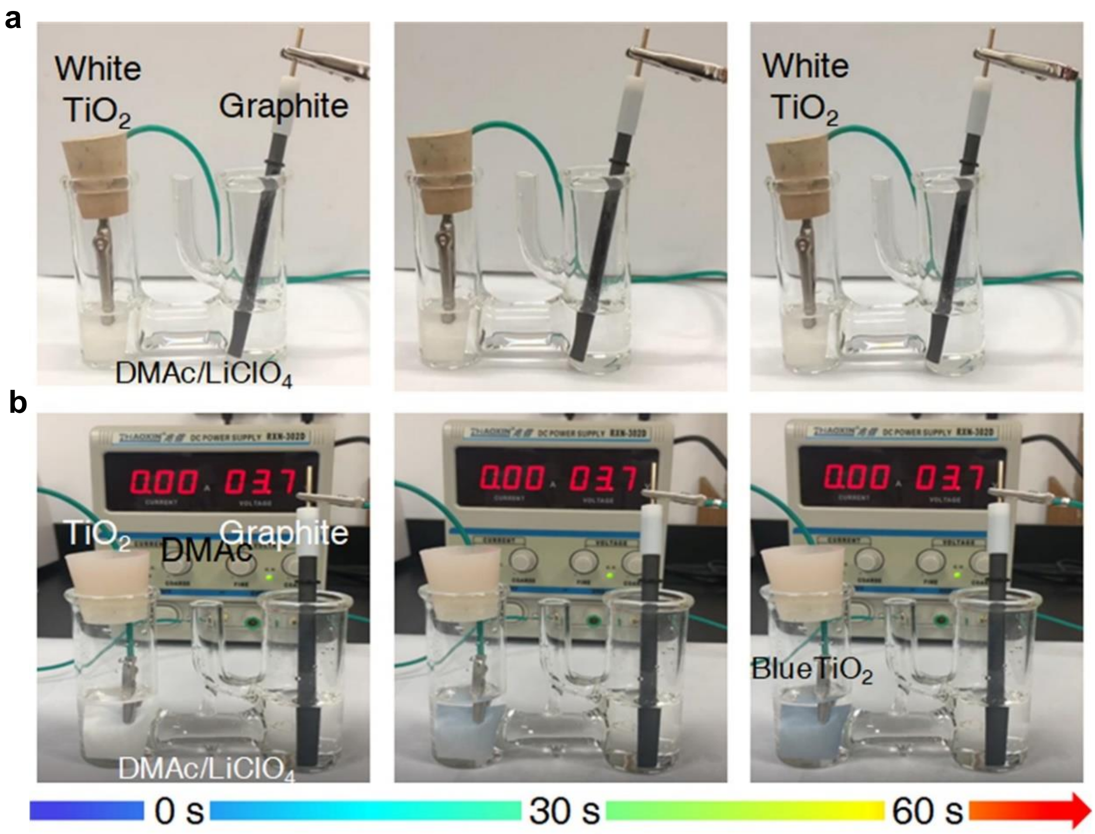

Supplementary Fig. 7. Visual display of the control experimental using graphite as the counter electrode to replace the initial Li-metal electrode. For both control experimental, the solvents were the same of DMAc/$LiClO_4$, but the difference was that there was a naked wire to connect the two films in (**a**), and there was an external voltage supplier of 3.7 V in (**b**). The white TiO_2 NF film did not change in (a) and it quickly changed to blue in 1 min in (b). Since there was not an obvious voltage difference between graphite and TiO_2, there was almost no electron flow in (a), thus confirming that the electron flow was also a necessary condition for the intercalation.

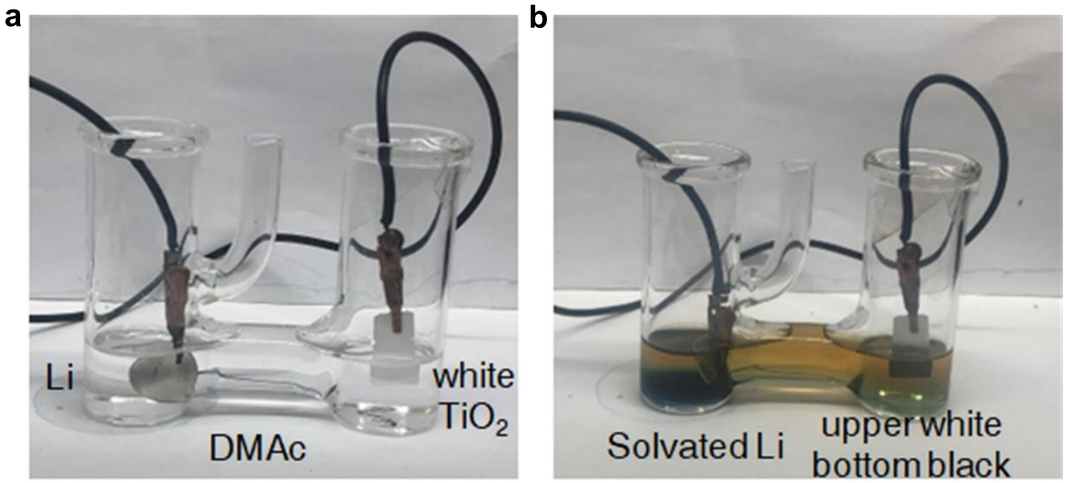

Supplementary Fig. 8. Demonstration of an evolving model from model 5. In this new derived model, only the bottom half of the TiO_2 NF film was immersed in the DMAc solvent, and the upper part was exposed in the air. As can be seen from Fig. 5b that only the soaked part of the film changed from white to black after 12 h, indicating that the solvated Li/Li^+ was a necessary condition for this intercalation.

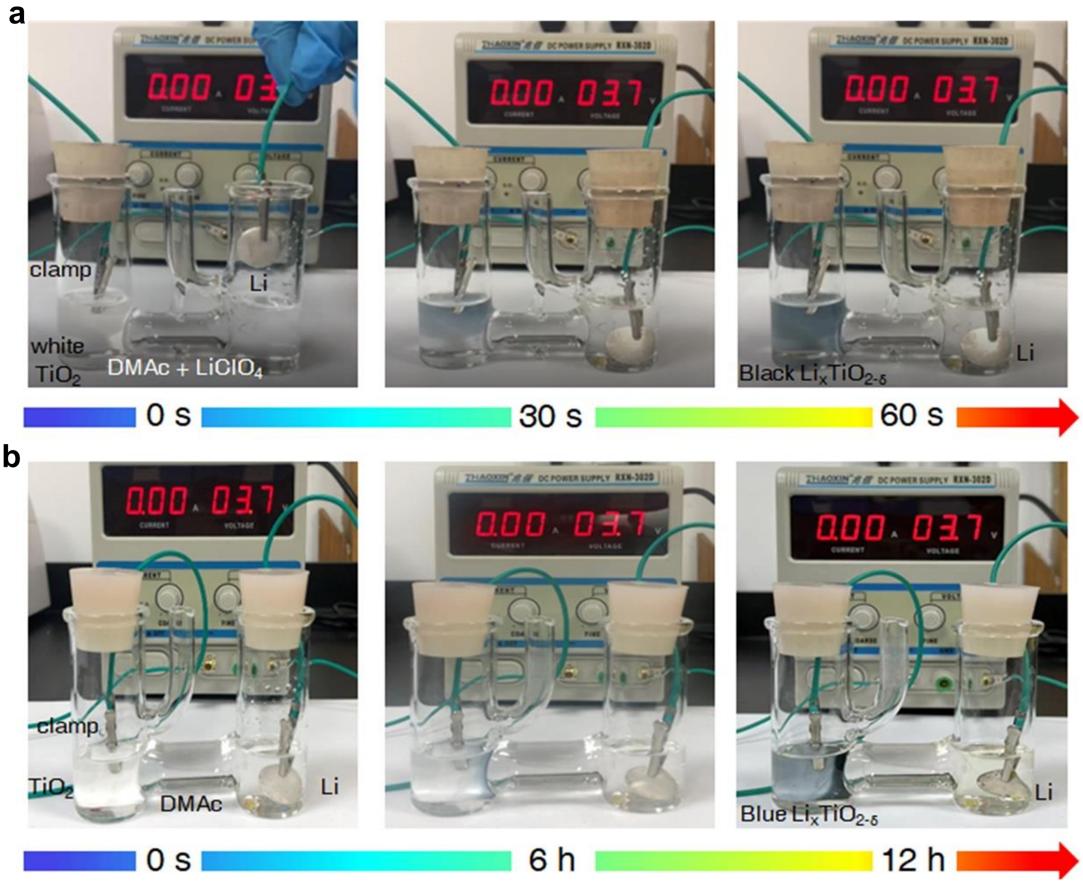

Supplementary Fig. 9. Visual display of experimental models 4 and 5. (**a**) Visualization of the color change process of the white TiO_2 NF film in model 5. The white film quickly turned into black in 1 min. (**b**) Visualization of the color change process of the white TiO_2 NF film in model 4. Due to the limited Li^+-concentration in the DMAc solvent, the intercalation kinetics was obviously decreased, and the white film turned into blue after 12 h. In addition, the white TiO_2 NF film started to turn blue at the side close from the Li-metal after 6 h.

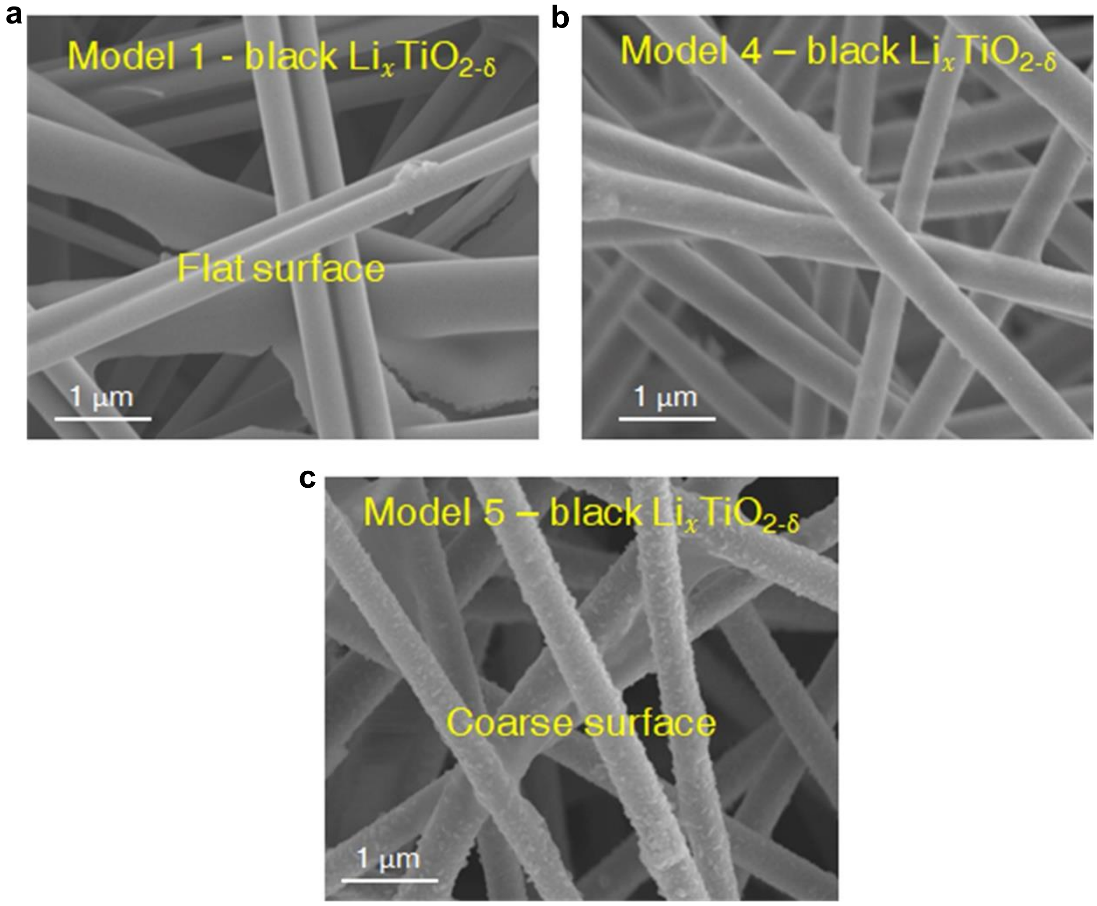

Supplementary Fig. 10. SEM images of the prepared topological $Li_xTiO_{2-\delta}$ NFs in (**a**) model 1, (**b**) model 4 and (**c**) model 5. There were obvious roughness differences in these three models, in which the NFs in model 5 show the coarsest surface that contained lots of particles.

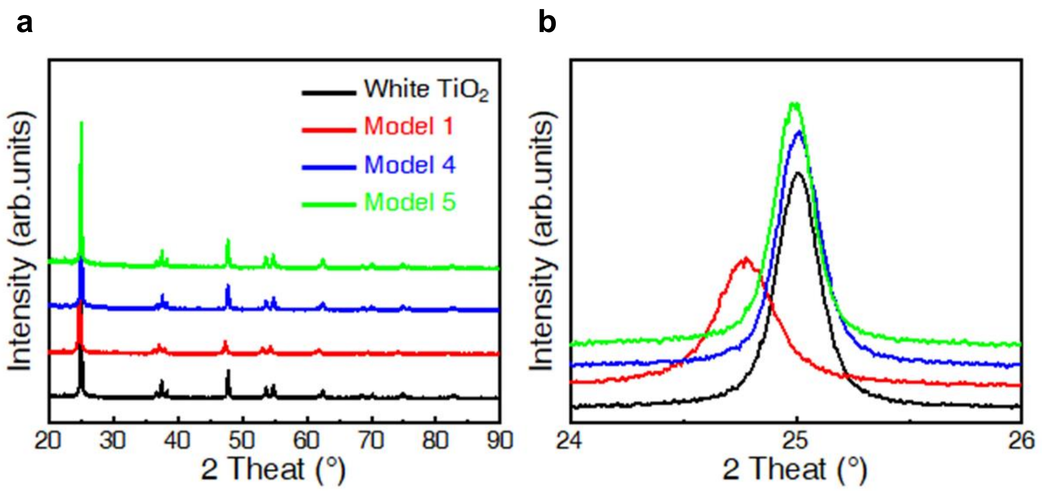

Supplementary Fig. 11. XRD patterns of different samples. (**a**) XRD patterns of the initial white TiO_2 NF film and the obtained intercalated NF films in model 1, model 4 and model 5. (**b**) Comparison of the peak shifts of these four samples at the range of 24-26°. The characteristic peak shift of TiO_2 obtained from model 1 was the largest, indicating that the contact intercalation reaction was the most rapid and intense.

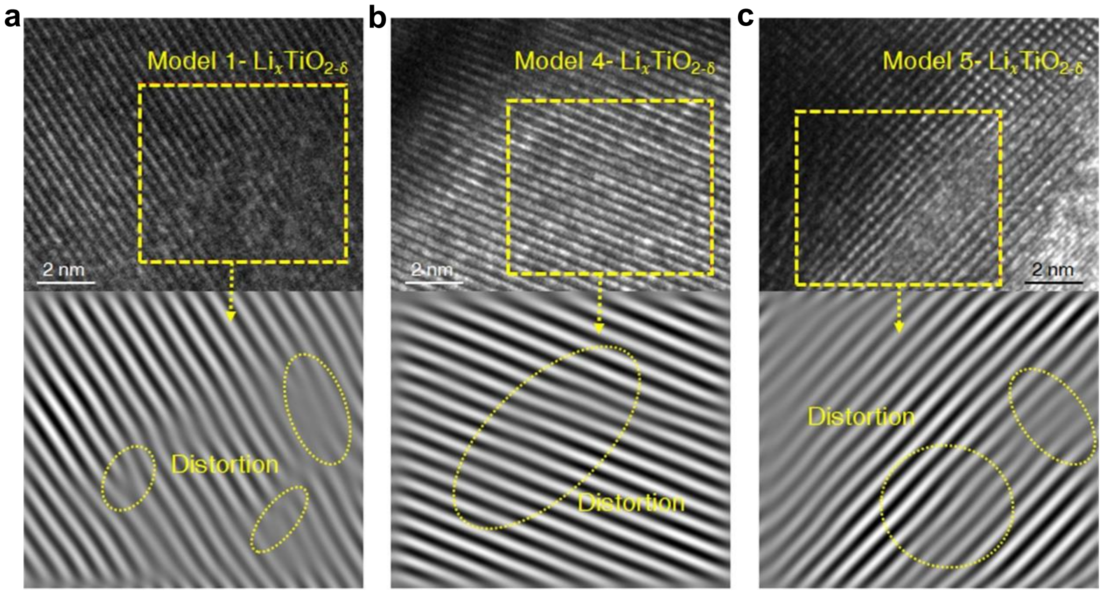

Supplementary Figure 12. TEM images and inverse FFT images of the as-prepared $Li_xTiO_{2-\delta}$ NFs by (**a**) model 1, (**b**) model 4, and (**c**) model 5. All these samples show lattice distortions after the intercalation reactions, indicating that the intercalation changed the crystal structures of TiO_2, and formed new metastable structure phases.

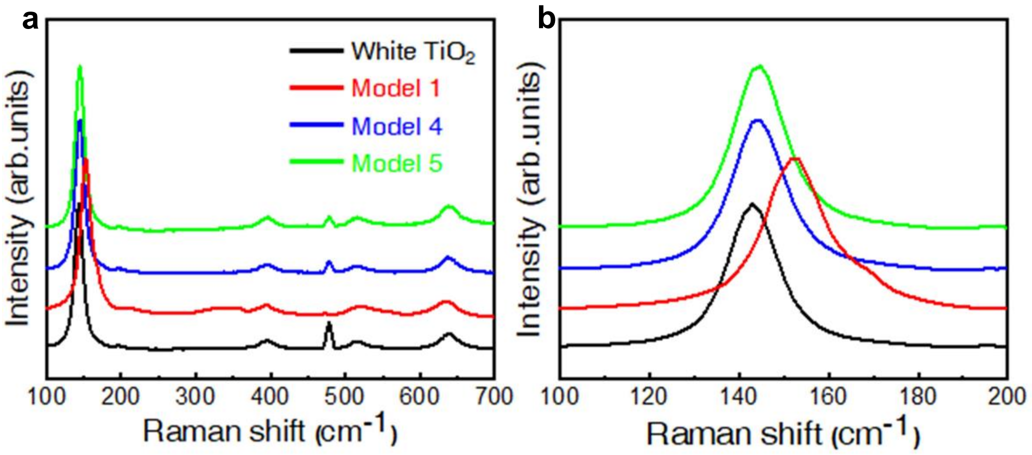

Supplementary Fig. 13. Raman characterization of different samples. (**a**) Raman characterization of the intercalated $Li_xTiO_{2-\delta}$ NF structures prepared by model 1, model 4, and model 5. (**b**) Comparison of the peak shifts of these four samples at the range of 100-200 cm^{-1}. The white TiO_2 NFs show six Raman-active modes, and obvious blue shift and broadened E_g peaks are found after the Li^+- intercalation due to the lattice distortions of TiO_2.

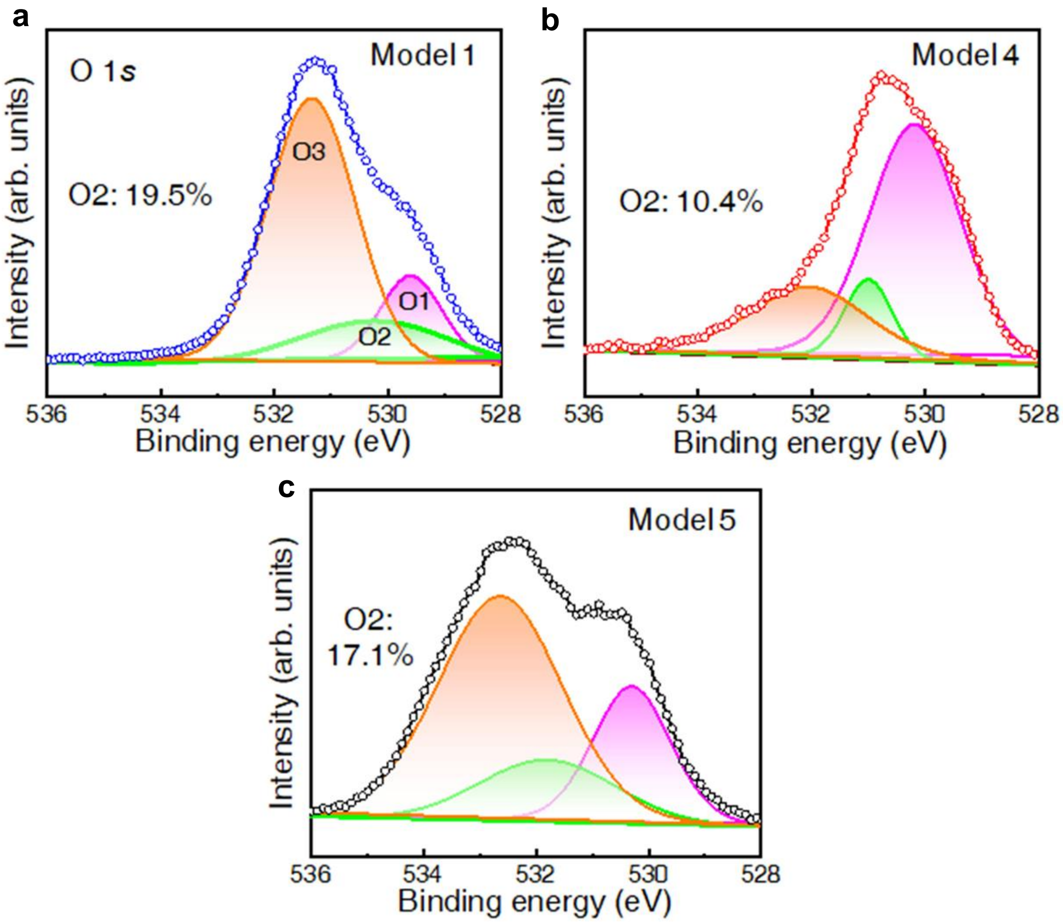

Supplementary Fig. 14. High-resolution XPS spectra of O 1*s* of different intercalated $Li_xTiO_{2-\delta}$ structures in (**a**) model 1, (**b**) model 4, and (**c**) model 5. Roughly calculations of the O2 peak intensity were obtained to compare the defect intensity of these three samples after the topotactic reaction.

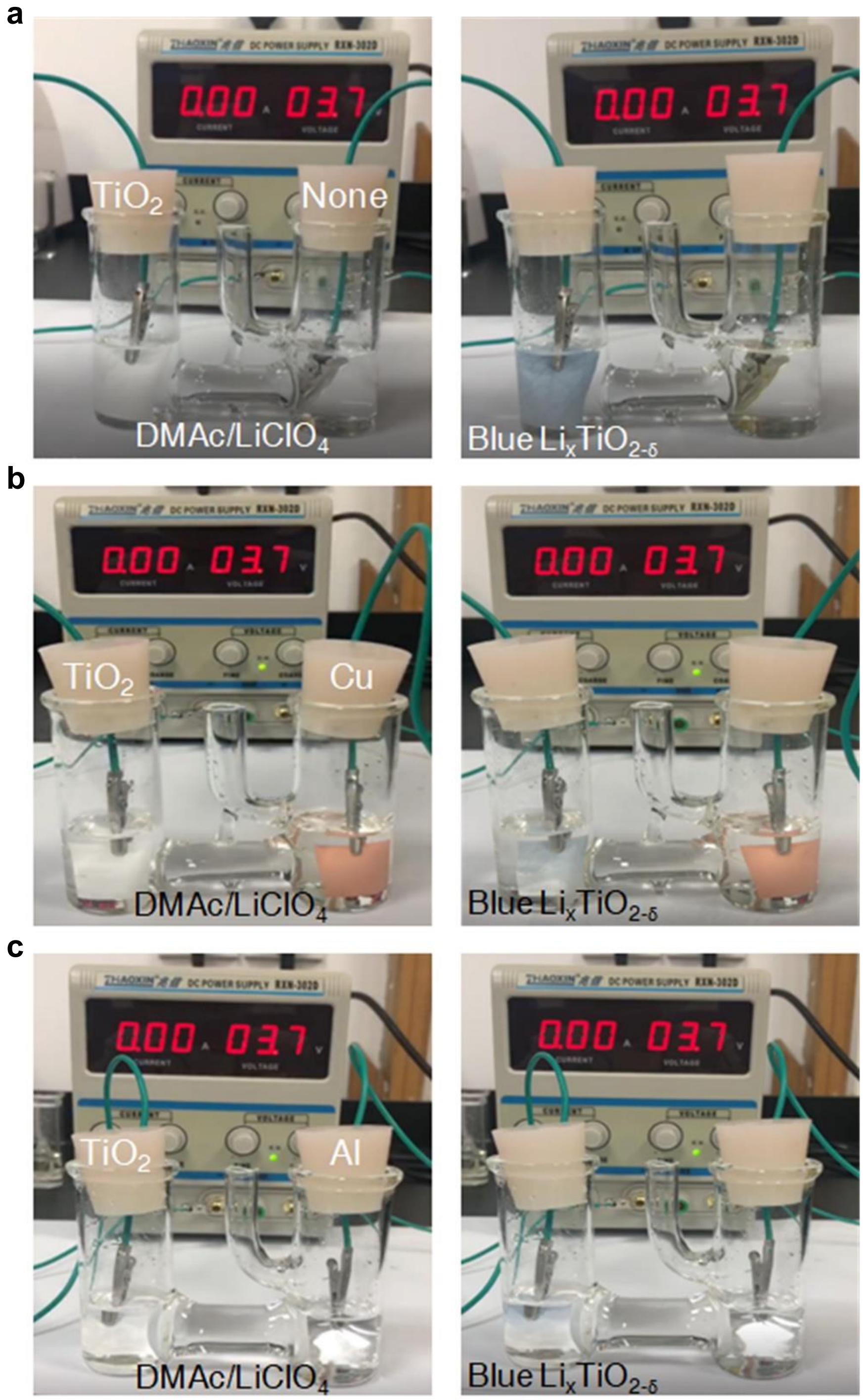

Supplementary Fig. 15. Visual display of the control experimental using different counter electrodes but the same solvent of DMAc/$LiClO_4$. Visualization of the color change process of the white TiO_2 NF film in the systems that (**a**) the counter electrode was conductive clip, (**b**) the counter electrode was copper foil, and (**c**) the counter electrode was aluminum foil. All the white TiO_2 NF films turned into blue in these three models.

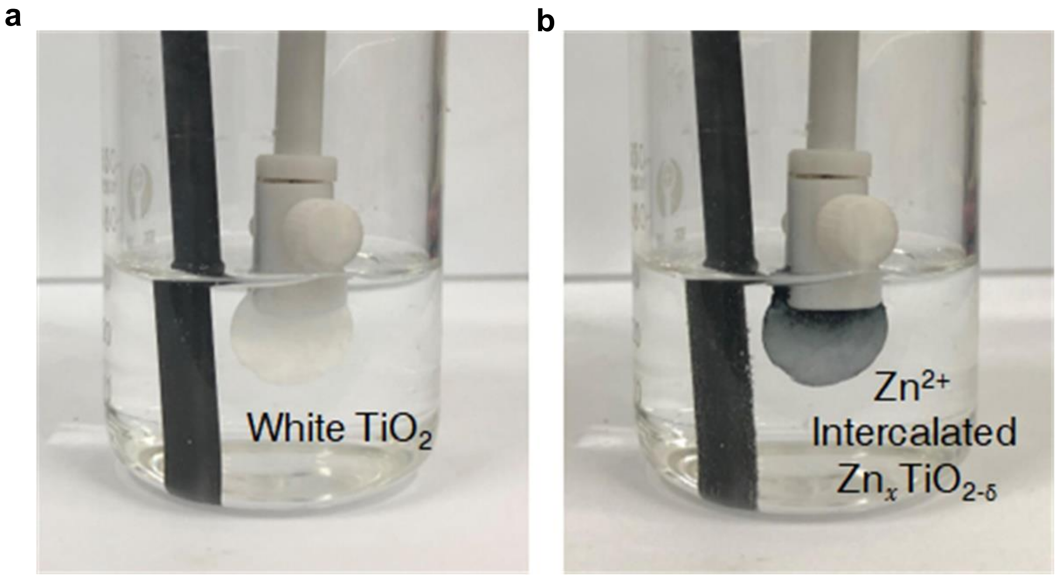

Supplementary Fig. 16. The intercalation of Zn^{2+}-ions into TiO_2 NFs with the model 4 prototype. Visualization of the color change process of the white TiO_2 NF film in a new model, in which the solvent contained 1M Zn^{2+}. When a small voltage of 3.7 V was applied, the white TiO_2 NF film was intercalated by Zn^{2+}, and a color change was observed visually. The solvent used in the experimental was DMAc + $Zn(NO_3)_2$.
