# Supplementary material for: Control of metal oxides’ electronic conductivity through visual intercalation chemical reactions
Source: Nat Commun. 2023 Oct 2;14:6130. doi: 10.1038/s41467-023-41935-x (PMC10545781; doi:10.1038/s41467-023-41935-x)
Supplement: Supplementary file 3 — Description of Additional Supplementary Files [file 41467_2023_41935_MOESM3_ESM.docx]

**Description of Additional Supplementary Files**

Supplementary Movie 1.

The topochemical synthetic processes of conductive black Li*_x_*TiO_2-δ_ NF films using model 1.

Supplementary Movie 2.

The topochemical synthetic processes of conductive black Li*_x_*TiO_2-δ_ NF films using model 3.

Supplementary Movie 3.

The display of soft TiO_2_ NF film.

Supplementary Movie 4.

The topochemical synthetic processes of conductive black Li*_x_*TiO_2-δ_ NF films using model 5.
